# Molecular detection of Indian Ocean Lineage Chikungunya virus RNA in field collected *Culex quinquefasciatus* Say from Bangkok, Thailand but no evidence of virus replication

**DOI:** 10.1371/journal.pone.0246026

**Published:** 2021-01-28

**Authors:** Atchara Phumee, Proawpilart Intayot, Sriwatapron Sor-suwan, Akanitt Jittmittraphap, Padet Siriyasatien

**Affiliations:** 1 Department of Medical Technology, School of Allied Health Sciences, Walailak University, Nakhon Si Thamarat, Thailand; 2 Medical Science Program, Faculty of Medicine, Chulalongkorn University, Bangkok, Thailand; 3 Vector Biology and Vector Borne Disease Research Unit, Department of Parasitology, Faculty of Medicine, Chulalongkorn University, Bangkok, Thailand; 4 Department of Microbiology and Immunology, Faculty of Tropical Medicine, Mahidol University, Bangkok, Thailand; CEA, FRANCE

## Abstract

Following an outbreak of chikungunya virus (CHIKV) infections in Thailand in 2019, numerous cases of CHIKV infection have been diagnosed in Bangkok, the capital of the country. In our previous investigation of the vectors for disease transmission, we found natural infection of CHIKV in both male and female *Aedes aegypti* mosquitoes collected from the outbreak areas in Bangkok. Some reports mentioned the detection of CHIKV in *Culex* mosquitoes. In Thailand, the *Culex quinquefasciatus* Say mosquito is a common species found in urban and rural settings that coexists with *Ae*. *aegypti*. However, the role of *Cx*. *quinquefasciatus* mosquitoes in the spread of the Indian Ocean Lineage (IOL) of CHIKV in Thailand has never been investigated. In this study, *Cx*. *quinquefasciatus* were collected (16 males and 27 females) from an outbreak area in Bangkok. Eight of the 27 in field-caught female *Cx*. *quinquefasciatus* were positive for IOL CHIKV RNA, and 99–100% identity and full 100% coverage of sequences similar to CHIKV isolated from female *Ae*. *aegypti* in Bangkok, Thailand, whereas viral RNA was not detected in male samples using nested-RT-PCR. To determine whether CHIKV is able to replicate in *Cx*. *quinquefasciatus*, the laboratory strain of *Cx*. *quinquefasciatus* was allowed to feed on blood containing IOL CHIKV isolated from patient serum. The nested-RT-PCR, virus isolation, and immunofluorescence assay (IFA) were performed for CHIKV detection and replication. The results showed that CHIKV RNA was detected in *Cx*. *quinquefasciatus* until day 4 post infection. CHIKV did not produce any remarkable signs of infection, dissemination, or transmission in *Cx*. *quinquefasciatus*, and cytopathic effect (CPE) was not observed in C6/36 cells when infected with supernatant obtained from *Cx*. *quinquefasciatus* at days 7, 10, 14, and 21 post infection when compared to *Ae*. *aegypti*. The data from this study infer that CHIKV may be detected in *Cx*. *quinquefasciatus* but that the mosquito is not able to transmit CHIKV in Thailand.

## Introduction

Chikungunya virus (CHIKV) is an emerging and re-emerging infection that has occurred in many countries over several continents, including the Americas, eastern Africa, the eastern Indian Ocean islands, the western Indian Ocean islands, France, Italy, India, Singapore, Malaysia, Indonesia, and Thailand [1, 2]. In Thailand, the reported of CHIKV outbreaks in the southern part of the country from the Bureau of Epidemiology, Department of Disease Control, Ministry of Public Health in 2008–2009 were affected more than 54,000 cases and the genetic studies found a characteristic alanine-to-valine mutation at amino acid residue 226 (A226V) of the *E1* envelope glycoprotein of an East/Central/South/Africa (ECSA) genotype [3]. In 2013, from 109 blood samples of suspected patients obtained from Bueng Kan province, northeastern region of Thailand, 51 samples had evidence of CHIKV infection. Genetic characterization with 4 samples of this outbreak showed that CHIKV was belonged to the Indian Ocean Lineage (IOL) with A226V of an ECSA genotype [4]. Recently, outbreaks of CHIKV in 2020 have spread rapidly in Thailand, especially in metropolitan Bangkok, the capital of the country. The Bureau of Epidemiology, Ministry of Public Health, Thailand, described that a total of 10,905 cases of CHIKV infection were reported from 72 out of 77 provinces in 2020 [5]. Interestingly, 1,061 cases of CHIKV infection were investigated from Bangkok during this outbreak because only a few cases of CHIKV infection have been reported in Bangkok over the past decade. Chansaenroj et al. (2020) demonstrated that the complete genome sequences of CHIKV isolated from patients and an *Aedes aegypti* mosquito from this outbreak in southwest Bangkok belonged to an East/Central/South African (ECSA) lineage, with a mutation at A226V. However, the outbreak of CHIKV in 2019 was grouped into the ECSA genotypes but showed differences strains from previous outbreaks in Thailand in 2008–2009 and 2013 [6]. The *Ae*. *aegypti* mosquito is considered the main vector of CHIKV in this outbreak [7]. In addition, CHIKV RNA has been detected in both sexes of the vector mosquitoes, *Ae*. *aegypti* (L.) and *Ae*. *albopictus* Skuse [8]. Chompoosri et al. (2016) proved that IOL CHIKV could be transmitted vertically in both *Ae*. *aegypti* and *Ae*. *albopictus* under laboratory conditions up to F5 and F6 generations, respectively by using one-step qRT-PCR and CHIKV isolation in LLC-MK2 cell line [9]. However, several reports showed that CHIKV can be detected in *Culex* spp. mosquitoes. For example, glycerinated *Culex* mosquitoes in Newala district of Tanganyika [10] and *Cx*. *quinquefasciatus* in Reunion Island [11] have been found infected with CHIKV by virus isolation in C6/36 cells, RT-PCR, and sequencing. CHIKV has been isolated from female ornithophilic *Cx*. *ethiopicus* Edwards collected from an African field study [12, 13]. In contrast, *Cx*. *p*. *quinquefasciatus* are not able to transmit the virus experimentally, with no infection detected in 19 mosquitoes by feeding on viremic vervet monkeys [14]. However, no studies have investigated the transmission capacity of Thai *Cx*. *quinquefasciatus* for CHIKV. Therefore, we studied *Cx*. *quinquefasciatus* from outbreak in Bangkok 2019 in the same area of CHIKV-positive male and female *Ae*. *aegypti*. The *Cx*. *quinquefasciatus* mosquito coexists with *Ae*. *aegypti* in urban and rural areas and may contribute to CHIKV transmission. In addition, wild-caught *Cx*. *quinquefasciatus* were evaluated for CHIKV using a nested-RT-PCR assay targeting the conserved *E1* envelope protein genes, and blood-fed mosquitoes were evaluated by PCR. Finally, the replication of CHIKV was studied in laboratory strains of *Cx*. *quinquefasciatus* by using nested-RT-PCR, virus isolation in C6/36 cells, and immunofluorescence assay.

## Materials and methods

### Ethics statement

The study was approved by the animal research ethics committee of Chulalongkorn University and adhered to the Animal Care and Use Protocol (CU-ACUP). The Faculty of Medicine, Chulalongkorn University, Bangkok, Thailand (COA No. 011/2563) approved this study, which abided by the Animals for Scientific Purposes Act and all relevant institutional policies and regulations regarding animal care and use at Chulalongkorn University. The use of hazardous agents was only initiated after approval from the institutional animal care and use committee (IACUC), Institutional Biosafety Committee (IBC), and/or Environmental Health and Safety Department. The use of human sample was approved by the Institutional Review Board of the Faculty of Medicine, Chulalongkorn University, Bangkok, Thailand (IRB no. 452/58), and the study was conducted in compliance with the international guidelines for human research protection as stated in the Declaration of Helsinki, The Belmont Report, the Council for International Organizations of Medical Sciences (CIOMS) guidelines and the International Conference on Harmonization in Good Clinical Practice (ICH-GCP) and 45CFR 46.101(b). The study was explained to participant, and informed consent was signed.

### Mosquito collection

A total of 43 adult mosquitoes (16 males and 27 females) were collected in and around the houses of confirmed CHIKV-infected patients at Chom Thong District, Bangkok, using a mosquito aspirator (Bioquip, USA). The mosquito collection was permitted from the Institute for Urban Disease Control and Prevention, Department of Disease Control, Ministry of Public Health, Bangkok, Thailand. The live mosquito samples were transferred to the laboratory of Vector Biology and Vector-Borne Disease Research Unit, Department of Parasitology, Faculty of Medicine, Chulalongkorn University. Based on their morphological characteristics, all samples were differentiated according to their sex and species.

### CHIKV detection in field-caught *Cx*. *quinquefasciatus* by nested-RT-PCR

Individual whole mosquitoes were distinguished based on species and sex and were placed in 1.5 ml tubes with 1X phosphate-buffered saline (PBS). Each mosquito sample was ground in 300 μl of 1XPBS and the samples were centrifuged at 11000 g for 5 min. Then, 200 μl of the supernatant was mixed with 200 μl of 2X Minimum Essential Medium Eagle (MEM) medium (HyClone, USA) containing 2% heat-inactivated FBS (Gibco, USA), 2% penicillin (100 U/ml) and streptomycin (100 μg/ml) (Sigma-Aldrich, USA) for virus isolation, and the pellet was processed for viral RNA extraction using the Invisorb Spin Virus RNA Mini viral RNA extraction kit (STRATEC Molecular GmbH, Germany) according to the manufacturer’s protocol.” The extracted RNA samples were used immediately for CHIKV detection at the *E1* region of the envelope protein genes, and the samples were maintained for long-term storage at -80°C. Virus detection was performed by nested-RT-PCR as described by Intayot et al. (2019) [7]. In brief, the first amplification was performed using the Superscript III one-step RT-PCR kit (Invitrogen, USA) in a final volume of 25 μl of reaction mixture, containing 12.5 μl of 2X reaction mix, 0.7 μl of 10 μM each primer, 1 μl of SuperScript III RT/Platinum *Taq* Mix, 4.1 μl of sterile nuclease-free water, and 6.0 μl of RNA template. The reaction began with initial denaturation at 50°C for 30 min, denaturation at 95°C for 15 min, and followed by 40 cycles of 95°C for 1 min, 64°C for 1 min, 72°C for 1 min, and the final extension at 72°C for 10 min. The second round amplification, 2 μl of the first amplification step were amplified with the different set of primers in a final volume of 25 μl, consisted of 2.5 μl of 10X buffer, 2.5 μl of 25 mM MgCl_2_, 2.5 μl of 2 mM dNTP, 0.4 μl of 10 μM each primer, 0.2 μl of 1 U *Taq* polymerase (Thermo Fisher Scientific, USA), and 14.5 μl of sterile nuclease-free water. The reaction mixture was incubated at 95°C for 3 min, followed by 40 cycles of 95°C for 30 sec, 62°C for 30 sec, 72°C for 1 min, and the final step at 72°C for 7 min. The amplified PCR products were electrophoresed through a 1.5% agarose gel, stained with ethidium bromide, and then visualized with Quantity one Quantification Analysis Software Version 4.5.2, Gel Doc EQ System (Bio-Rad, USA).

### DNA cloning and sequencing

The PCR amplicons of CHIKV were directly ligated to pGEM-T Easy Vector (Promega, USA) using T4 DNA ligase, following the manufacturer's protocols. The recombinant DNA were transformed into competent of *Escherichia coli* DH5α cell and then screened the bacterial transformants using the blue-white colony system. The colonies suspected to carry the DNA fragment were cultured, and the plasmid DNA was isolated using the Invisorb Spin Plasmid Mini Kit (STRATEC molecular GmbH, Germany) according to the manufacturer’s instructions. The purified plasmids were sent to a commercial company (Macrogen, South Korea) for direct DNA sequencing. Nucleotide sequences were analyzed by comparison with the GenBank database using a BLAST program.

### Phylogenetic analysis

The nucleotide sequences were aligned using BioEdit Sequence Alignment Editor Version 7.2.5 [15]. Phylogenetic trees were generated using the maximum-likelihood method with IQ-TREE on the IQ-TREE web server (http://iqtree.cibiv.univie.ac.at/) with 1000 ultrafast bootstrap replicates. The best fit model of substitution was identified using the auto function on the IQ-TREE web server [16]. The phylogenetic tree was finally viewed and edited with FigTree v1.4.4 software (http://tree.bio.ed.ac.uk/software/figtree/).

### CHIKV infection of mosquitoes and virus detection in mosquito tissues

Indian Ocean Lineage (IOL) CHIKV isolated from a Thai patient during a recent outbreak in central (PCHIK2019) Thailand was used in this study. The procedure for virus propagation and virus titration in the C6/36 cell line followed the methods described by Potiwat et al. (2011) [17]. Laboratory colonies of *Ae*. *aegypti* (generation > F_250_) and *Cx*. *quinquefasciatus* (generation > F_300_) mosquitoes were maintained under standard conditions as follows: 28 ± 2°C, 65–85% relative humidity, and a 12/12-hour light/dark cycle. For CHIKV infection, 9.2×10^6^ PFU/ml of PCHIK2019 from expired human blood from deidentified donors [18], which was obtained from the National Blood Center, Thai Red Cross Society, Bangkok, Thailand, was used. Five-day-old starved females *Cx*. *quinquefasciatus* and *Ae*. *aegypti* were allowed to feed on the blood containing CHIKV through an artificial apparatus for 60 min. Engorged females were maintained in a cage provided with a 5% sucrose and 5% vitamin B complex (w/v). Two days after the infectious blood meal, a black plastic bowl containing water was placed into the mosquito cage for 3 days to allow for oviposition. Fourteen mosquitoes were dissected to collect the midguts, ovaries, and salivary glands at 7, 10, 14, and 21 days post infection (dpi) for evaluation using immunofluorescence assay (IFA). After dissection, other tissues were individually transferred to a 1.5 ml microcentrifuge tube with 300 μl of 1XPBS following the above protocol for RNA extraction and virus isolation.

### Virus isolation and propagation

Fifty microliters of each suspension were inoculated onto confluent monolayers of *Ae*. *albopictus* C6/36 cells (ATCC CRL-1660) for 1 hour in 24-well plates grown in minimum essential medium (MEM) (Sigma-Aldrich, USA) supplemented with 10% heat-inactivated fetal bovine serum (FBS) (Sigma-Aldrich, USA). The cultures were incubated at 37°C in 5% CO_2_ and monitored daily for cytopathic effects (CPE) at 7, 10, 14, and 21 dpi by nested-RT-PCR and IFA.

### Immunofluorescence assay (IFA)

After dissection in 1xPBS, midguts, ovaries, and salivary glands at 7, 10, 14, and 21 dpi were placed on SuperFrost Plus microscope slides (Thermo Scientific, USA). Midguts, ovaries, and salivary glands were fixed in 4% paraformaldehyde for 15 min, rinsed with 1xPBS and then incubated for 15 min in 0.1% of Triton X-100. They were washed again with 1xPBS (3x5 min). The slides were drained and incubated for 60 min with mouse monoclonal [B1412huM] (Abcam, MA) as the primary antibody at the concentration of 1:1000 and then washed with 1xPBS (3x5 min). After washing, all slides were then incubated for 60 min with 1: 5000 Goat Anti-Mouse IgG H&L (Fluorescein isothiocyanate: FITC) (ab6785) (Abcam, MA) as the secondary antibody and then washed with 1xPBS (3x5 min). Finally, a drop of Prolong gold antifade (Invitrogen, USA) was applied on each slide, and a cover slide was placed on top. The negative mosquitoes run as a negative control. All slides were examined under a fluorescence microscope (Nikon, Japan).

### Evaluation of CHIKV RNA detection in blood-fed laboratory *Cx*. *quinquefasciatus* strain

Female *Cx*. *quinquefasciatus* or *Ae*. *aegypti* were allowed to feed on blood containing CHIKV (PCHIK2019) at 9.2×10^6^ PFU/ml through an artificial feeding apparatus. Fully fed females were moved to a new cage and placed in an environmentally controlled room. Ten engorged mosquitoes were collected per day for virus detection. Individual mosquitoes were ground in 300 μl of 1XPBS, and the pellet was processed for viral RNA extraction. Two hundred microliters of the supernatant were mixed with 200 μl of 2X MEM medium and used for virus isolation. Mosquitoes were evaluated for CHIKV using nested-RT-PCR every day until the CHIKV RNA could not be detected in *Cx*. *quinquefasciatus*.

## Results

### CHIKV detection and isolation in field-caught *Cx*. *quinquefasciatus*

We conducted CHIKV surveillance in mosquitoes collected in and around houses of confirmed CHIKV-infected patients at Chom Thong District, Bangkok, Thailand. A total of 43 *Cx*. *quinquefasciatus* adults were collected (16 males and 27 females) and *Ae*. *aegypti* were reported by Intayot et al. (2019) [7]. Eight females were positive for CHIKV RNA using nested-RT-PCR. Interestingly, all 8 positive samples showed undigested blood in the abdomen.

**Fig 1:**
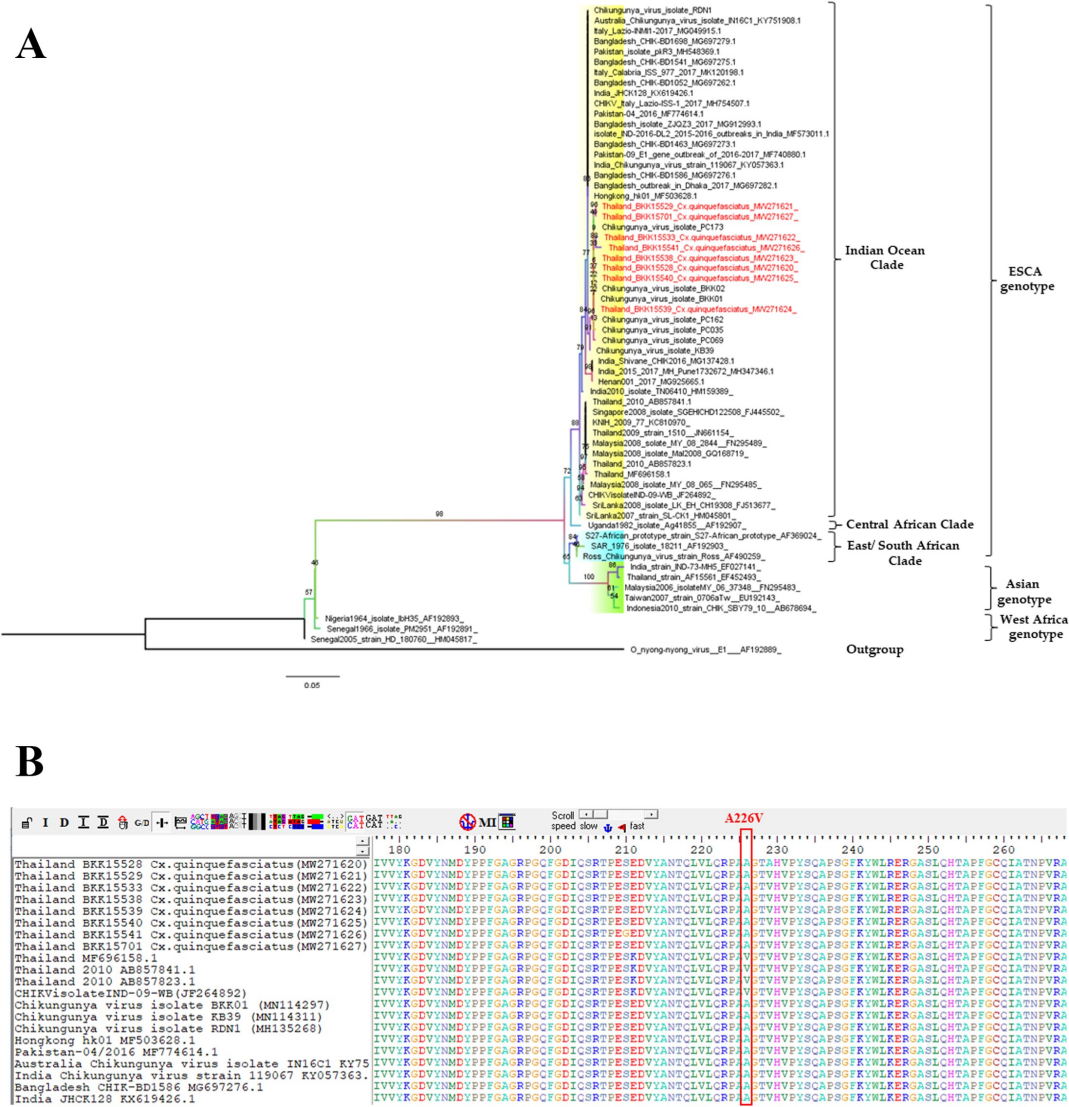
Phylogenetic tree of CHIKV in *Cx*. *quinquefasciatus* constructed from partial *E1* sequences. The maximum likelihood was constructed with IQ-TREE by using the maximum-likelihood method with 1000 ultrafast bootstrap replicates. The best-fit model of substitution was found using the auto function on the IQ-TREE web server. The sequences from this study are indicated with a red color (A) and showing portion of alignment of amino acid sequences of the *E1* gene of CHIKV in *Cx*. *quinquefasciatus* at position of the A226V mutation is indicated by a vertical column.

A total of 8 positive samples were performed the sequences. All sequences consisted of 539 nucleotides in length. The BLAST result showed that the sequence also similar to CHIKV isolated from female *Ae*. *aegypti* in Bangkok, Thailand with 99–100% identity and full 100% coverage of the nucleotide sequences (Accession no. MN114297). All sequences of CHIKV in *Cx*. *quinquefasciatus* were submitted to the GenBank database under accession numbers MW271620-MW271627. The result of phylogenetic tree revealed that these CHIKV sequences were classed into the Indian Ocean clade, which belonged to the ECSA genotype (Fig 1A); moreover, no presence of the *E1*: A226V in Indian Ocean clade of CHIKV showed in present study (Fig 1B). However, CHIKV isolation from the field-caught *Cx*. *quinquefasciatus* indicated no CPE at days 7, 10, and 14 in all positive samples. These findings suggested that there was insufficient evidence to conclude that there was no replication of CHIKV in field-caught *Cx*. *quinquefasciatus*. Therefore, we studied the evidence of IOL CHIKV infection in *Cx*. *quinquefasciatus* in laboratory conditions compared to *Ae*. *aegypti*.

### Demonstration of CHIKV replication and distribution in laboratory strain *Cx*. *quinquefasciatus* compared to *Ae*. *aegypti*

**Fig 2:**
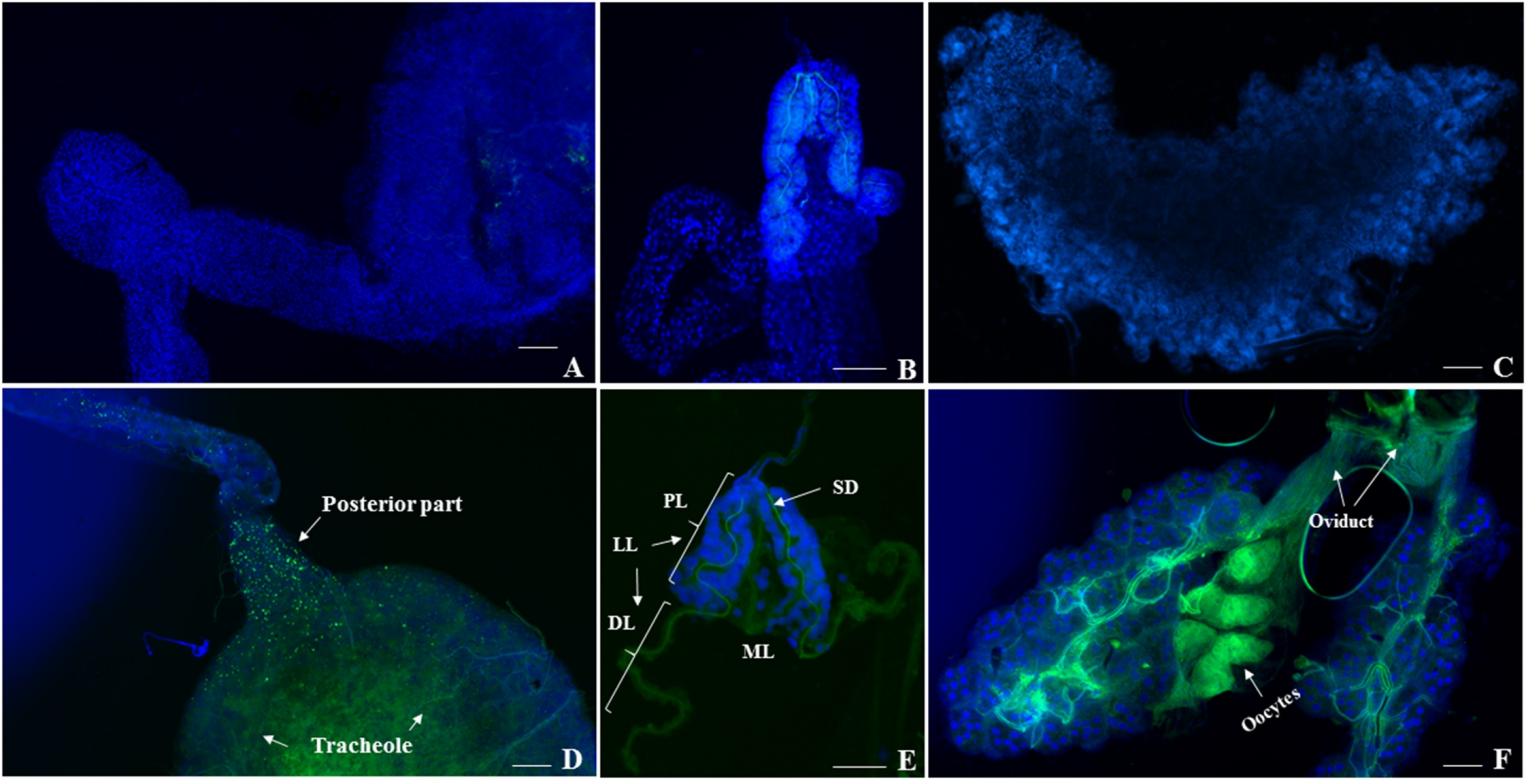
IFA detection of viral antigens in CHIKV at 10 dpi in midgut (A), salivary gland (B), and ovary (C) dissected from females *Cx*. *quinquefasciatus* and CHIKV infection in midgut (D), salivary gland (E), and ovary (F) of female *Ae*. *aegypti*. DAPI signal (blue) and CHIKV antigen (FITC/green), ML: Median lobe, LL: Lateral lobe, PL: Proximal region of lateral lobes, DL: Distal region of lateral lobes, SD: Salivary duct (Both midgut and ovary are shown at 100x magnification, and salivary gland is shown at 200x magnification).

**Fig 3:**
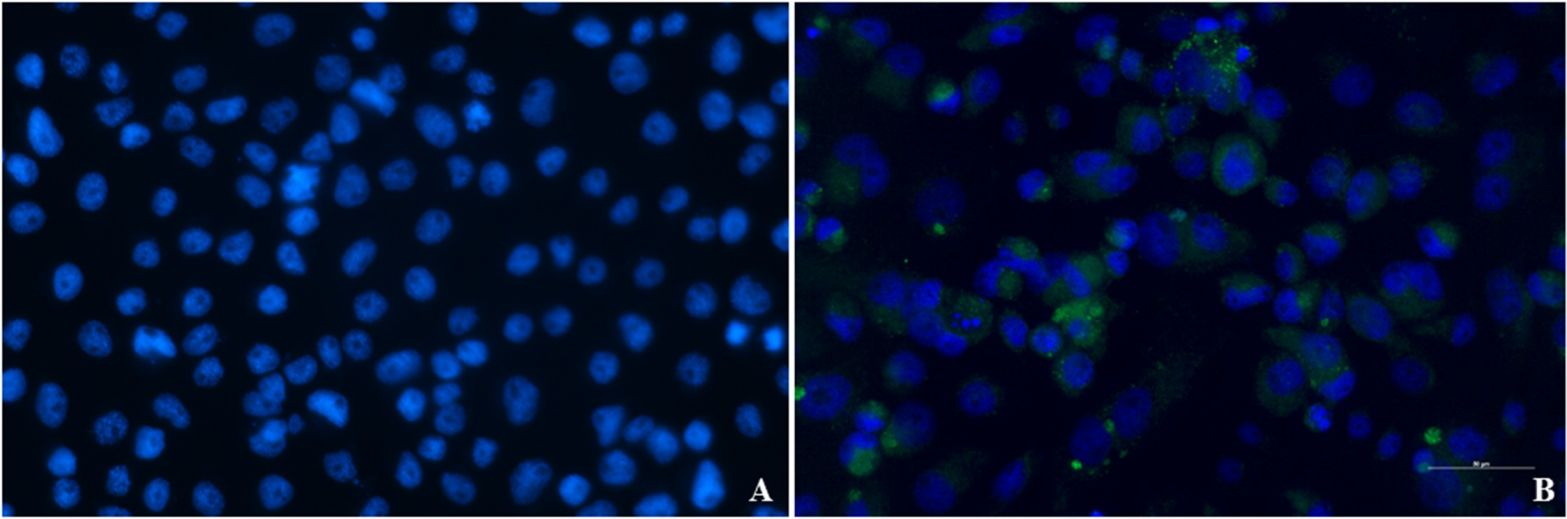
Replication of CHIKV in *Cx*. *quinquefasciatus* (A) and *Ae*. *aegypti* (B) in C6/36 cells. CHIKV was detected using IFA; green fluorescent color indicates viral replication, and nuclei were stained with DAPI (blue fluorescent color).

We investigated the replication and distribution of IOL CHIKV in midguts, ovaries, salivary glands, and other tissues of *Cx*. *quinquefasciatus* and *Ae*. *aegypti* on various days post infection (7, 10, 14, 21 dpi). Replication and distribution of CHIKV were not observed in the midguts (Fig 2A), salivary glands (Fig 2B), ovaries (Fig 2C), and other tissues of *Cx*. *quinquefasciatus* by nested-RT-PCR, virus isolation, and IFA. In contrast, CHIKV infection was detected in the midguts, ovaries, salivary glands, and other tissues of *Ae*. *aegypti* as early as 7 dpi by nested-RT-PCR, virus isolation, and IFA. The maximum infection rate (midgut) and dissemination rate (other tissues) were found at 14 dpi and 10 dpi, respectively. For IFA assay, we examined the presence of CHIKV antigen in the cytoplasm of midgut cells at the posterior part as well as in gut-associated tracheoles at 7–21 dpi (Fig 2D). For transmission, CHIKV was first clearly detected in lateral lobes and the median lobe of the salivary glands at 7–21 dpi by IFA. All distal lobes of salivary glands showed the presence of some disrupted CHIKV and showed CHIKV antigens in cells of the salivary duct (Fig 2E), whereas we could not detect morphological damage in the proximal part of the lateral lobes. While vertical transmission was not found in *Cx*. *quinquefasciatus*, CHIKV antigen was visible in cells of CHIKV-infected ovaries and oviducts of *Ae*. *aegypti*, and CHIKV infection was demonstrated in a small number of oocytes in ovaries at 10 dpi (Fig 2F). From the other tissues, we separated the supernatant with MEM medium for virus culture in C6/36 cells and examined them by IFA. The results revealed that no evidence of CHIKV replication or CPE in cells treated with supernatant from *Cx*. *quinquefasciatus* (Fig 3A), whereas cells treated with supernatant from *Ae*. *aegypti* showed CPE at 7dpi (Fig 3B).

### Evaluation of CHIKV detected in the laboratory *Cx*. *quinquefasciatus* strain after CHIKV infected blood feeding

At days 1–3 after infection, the undigested blood in the abdomen of each individual *Cx*. *quinquefasciatus* was observed. Interestingly, by nested-RT-PCR, CHIKV RNA in *Cx*. *quinquefasciatus* was detected until 4 dpi, although undigested blood was not observed in all samples. CPE appeared in C6/36 cells at day 1 after infection from 1/10 (10%) *Cx*. *quinquefasciatus* samples, but infections from *Ae*. *aegypti* samples showed CPE since day 1 after CHIKV infection in all samples until up to 21 days (Table 1). This study concludes that CHIKV particles are therefore digested by the *Cx*. *quinquefasciatus* alongside the actual blood meal and are not replicated in C6/36 cells. However, we found clear evidence of replication of CHIKV in *Ae*. *aegypti*.

**Table 1:** Detection and isolation of CHIKV in *Cx*. *quinquefasciatus* and *Ae*. *aegypti* by nested-RT-PCR and virus isolation.

	Nested-RT-PCR detection (%)										Virus isolation (%)									
Day post infection (dpi)	1	2	3	4	5	6	7	10	14	21	1	2	3	4	5	6	7	10	14	21
*Cx*. *quinquefasciatus*	100	100	70	20	0	0	0	0	0	0	10	0	0	0	0	0	0	0	0	0
*Ae*. *aegypti*	100	100	100	100	100	100	90	80	60	30	100	100	100	100	100	100	90	80	60	30

## Discussion

In 2019, CHIKV re-emerged in Thailand, especially Bangkok, the capital of the country. Studies have revealed the genetic variability of CHIKV from this outbreak in both patients [6] and *Ae*. *aegypti* mosquitoes [7]. Several reports mentioned that *Ae*. *aegypti* and *Cx*. *quinquefasciatus* are potential vectors for several diseases, such as Japanese encephalitis, chikungunya, Zika fever, West Nile fever, yellow fever, and filariasis [19, 20]. *Cx*. *quinquefasciatus* is widely distributed in urban and rural areas of Thailand and shares similar patterns of habitats with *Ae*. *aegypti* during their life cycle because they have aquatic and terrestrial stages [21]. In this study, we collected *Cx*. *quinquefasciatus* from the same outbreak area in Bangkok, 2019, where CHIKV was detected in male and female *Ae*. *aegypti* [6, 7]. Among 43 samples of *Cx*. *quinquefasciatus*, CHIKV RNA was detected in 8 female samples. We tried to demonstrate the viability of the virus in all nested-RT-PCR-positive samples through infection in C6/36 cells, but CPE was not observed. The supernatant collected from infected cells was also negative by nested-RT-PCR at different time-points. Several reports have suggested that *Culex* species clearly demonstrate the potential to be a vector for CHIKV, such as glycerinated *Culex* mosquitoes in Newala district of Tanganyika [10], *Cx*. *quinquefasciatus* in Reunion Island [11], and *Cx*. *ethiopicus* Edwards in an African field study [12, 13]. Recently, Ribeiro Cruz et al. (2020) revealed that CHIKV was isolated in pools of *Ae*. *aegypti* females and male and in *Cx*. *quinquefasciatus* females (n = 2); however, this report cannot speculate and assume that *Cx*. *quinquefasciatus* is participating in the CHIKV transmission. Moreover, the limitation of the study was not performed of salivary gland analysis, which could determine of vector competence [22]. In contrast, reports have shown evidence of a lack of competence of *Cx*. *p*. *quinquefasciatus* for CHIKV [14].

Laboratory studies were conducted with *Cx*. *quinquefasciatus* infected with IOL CHIKV isolated from a patient. The CHIKV isolate was not able to replicate in *Cx*. *quinquefasciatus* by virus isolation and IFA, and viral RNA was not detected by nested-PCR, in contrast to *Ae*. *aegypti* mosquitoes, which were used as a positive control. Moreover, we evaluated CHIKV in blood feed of female *Cx*. *quinquefasciatus* compared with *Ae*. *aegypti*. Undigested blood could be observed until 3 dpi in the abdomens of the mosquitoes, while CHIKV RNA in *Cx*. *quinquefasciatus* was detected until 4 dpi using nested-RT-PCR. In addition, CPE at day 4 was shown in 1 sample at day 1 after infection. There have been reports on the duration of blood nucleic acid detection in mosquitoes, such as 7 days post feeding in *Cx*. *tarsalis* Coquillett [23], up to 3 days after feeding in *Cx*. *p*. *pipiens L*. [24], only up to 2 days after feeding in *Anopheles stephensi* mosquito [25], and 3 days post feeding in *Ae*. *aegypti* [26]. However, the differences in the duration of detection of blood nucleic acid in mosquitoes depend on different digestive processes and mosquito species. We hypothesized that the positive results of CHIKV detection and CPE in *Cx*. *quinquefasciatus* during 1–4 dpi were due to the live virus remaining in the undigested infectious blood meal rather than CHIKV replication in *Cx*. *quinquefasciatus*.

Current virus detection techniques such as RT-PCR for the detection of viral genome, virus isolation or cell culture for infectious viral particle titration, and IFA for detection of viral antigen and for visualization and localization of target virus have been used for CHIKV detection in human samples [27]. In this study, we used *E1* nested-RT-PCR, virus isolation, and IFA techniques to demonstrate CHIKV in a mosquito cell line and in the mosquitoes. This modified IFA protocol is simple, rapid and versatile for demonstration of the virus antigen and viral growth in infected cell and mosquitoes. This technique can also confirm true CHIKV infection and replication in mosquitoes. The IFA can also be used to detect CHIKV shortly after the initiation of infection in the mosquito midgut and demonstrate the virus dissemination in the mosquitoes. Therefore, IFA can be used as a tool for investigating CHIKV transmission in mosquitoes.

Our studies provided information to better understand the vector competence for current circulating CHIKV. Our findings indicated that *Cx*. *quinquefasciatus* is not a competent vector of CHIKV in Thailand. We assumed that CHIKV-infected female *Cx*. *quinquefasciatus* in the field might be maintaining the virus in patient blood because mosquitoes were collected from active CHIKV infected patients' homes. A report from Florida demonstrated CHIKV infection in *Cx*. *p*. *quinquefasciatus*, but it showed poor vector competence under laboratory conditions, with no transmission and dissemination of CHIKV in the mosquito [28]. This finding has important implications, as CHIKV-infected mosquitoes may not be able to transmit the virus. McIntosh et al. (1963) suggested that CHIKV infection and transmission did not occur in *Cx*. *quinquefasciatus* [29]. However, several factors could have affected the competency of the mosquitoes for virus transmission, such as the genetic variations in vectors between mosquito species, virus strains, doses of the virus for infection, and mosquito immunity and defense mechanisms [30–32]. The current study suggests that we can detect CHIKV RNA from field-collected *Cx*. *quinquefasciatus*, but transmission of virus does not occur in *Cx*. *quinquefasciatus* strains from Thailand. This *Cx*. *quinquefasciatus* species does not have competency to transmit CHIKV in either field or laboratory conditions. The real mechanism for why CHIKV is not able to replicate in *Cx*. *quinquefasciatus* should be investigated in the future for an improved understanding of virus-vector interaction.
